# Inter-atrial septal dehiscence due to incomplete closure of trans-septal incision after cardiac surgical bi-atrial trans-septal approach

**DOI:** 10.1093/ehjci/jead226

**Published:** 2023-09-07

**Authors:** Gabriella Locorotondo, Christian Colizzi, Antonella Lombardo, Massimo Massetti

**Affiliations:** Department of Cardiovascular Sciences, Fondazione Policlinico Universitario A. Gemelli IRCCS Rome, Largo A. Gemelli 8, 00168 Rome, Italy; Department of Cardiovascular Sciences, Fondazione Policlinico Universitario A. Gemelli IRCCS Rome, Largo A. Gemelli 8, 00168 Rome, Italy; Department of Cardiovascular Sciences, Fondazione Policlinico Universitario A. Gemelli IRCCS Rome, Largo A. Gemelli 8, 00168 Rome, Italy; Department of Cardiovascular Sciences, Catholic University of the Sacred Heart, Rome, Italy; Department of Cardiovascular Sciences, Fondazione Policlinico Universitario A. Gemelli IRCCS Rome, Largo A. Gemelli 8, 00168 Rome, Italy; Department of Cardiovascular Sciences, Catholic University of the Sacred Heart, Rome, Italy

**Figure:**
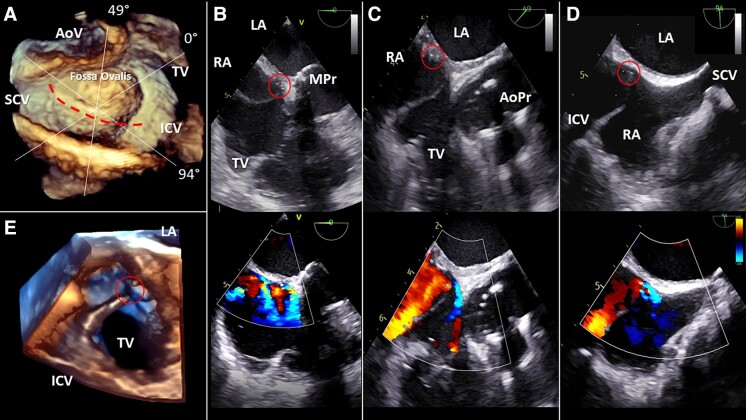


A 70-year-old woman underwent surgical replacement of aortic valve and mitral valve by bio-prostheses and annuloplasty of tricuspid valve due to rheumatic disease. A bi-atrial trans-septal approach (*Panel A*, dotted red line) was used, and inter-atrial septal (IAS) incision was finally closed with a 4-0 prolene suture. Post-operative transthoracic echocardiogram (TTE) revealed a residual mild left-to-right shunt at the level of IAS. During the following years, the patient remained asymptomatic. About 7 years after cardiac surgery, TTE showed an increase of inter-atrial shunt. Transoesophageal echocardiography (TEE) revealed intimal layer debris on the right side of IAS (*Panel B*, upper; see Supplementary data online, *Videos S1* and *S2*), expanding into the right atrium (RA). A significantly prominent eustachian valve was involved by dehiscence at its insertion point on the IAS (*Panels C* and *D*, upper; see Supplementary data online, *Videos S3–S5*). Small hyperechoic points (red circles) denoted the residual of stitches. Colour Doppler mode revealed left-to-right shunt originating from multiple sites, probably along the suture line (*Panel B*, bottom; see Supplementary data online, *Video S6*), and exiting into the RA above the eustachian valve (*Panels C* and *D*, bottom; see Supplementary data online, *Videos S7* and *S8*). Whether the blood flowing from inferior cava vein towards IAS might have contributed to damage IAS at a ‘locus minoris resistentiae’ is unknown. This is the first report of IAS dehiscence as a consequence of incomplete closure of bi-atrial trans-septal incision. TEE is essential to formulate diagnosis, and volumetric rendering (*Panels A* and *E*; see Supplementary data online, *Video S9*) allows to better understand the spatial relationships between cardiac structures.

Supplementary data are available at *European Heart Journal - Cardiovascular Imaging* online.

**Funding:** None declared.

**Data availability:** The data underlying this article will be shared on reasonable request to the corresponding author.

## Supplementary Material

jead226_Supplementary_Data

